# Supplementary material for: DELE1 tracks perturbed protein import and processing in human mitochondria
Source: Nat Commun. 2022 Apr 6;13:1853. doi: 10.1038/s41467-022-29479-y (PMC8986780; doi:10.1038/s41467-022-29479-y)
Supplement: Supplementary file 1 — Supplementary Information [file 41467_2022_29479_MOESM1_ESM.pdf]

SUPPLEMENTARY INFORMATION

DELE1 tracks perturbed protein import and processing in human mitochondria

Evelyn Fessler[1,*], Luisa Krumwiede[1] & Lucas T. Jae[1,*]

[1]Gene Center and Department of Biochemistry, Ludwig-Maximilians-Universität München, Feodor-Lynen-Strasse 25, 81377, Munich, Germany.

[*]Evelyn Fessler fessler@genzentrum.lmu.de
[*]Lucas T. Jae jae@genzentrum.lmu.de

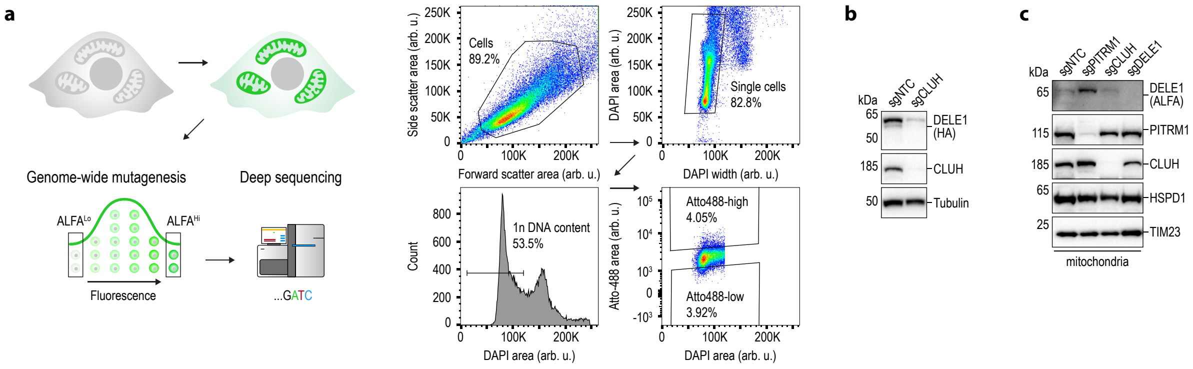

Supplementary Figure 1. PITRM1 and CLUH regulate the levels of DELE1.

a Schematic depicting work flow of haploid genetic screen for regulators of DELE1 and flow cytometry gating strategy. Arb. u., arbitrary units.

b HeLa *DELE1*^{HA} cells exposed to the indicated sgRNAs were analyzed by immunoblotting.

c HAP1 *DELE1*^{ALFA} cells were exposed to the specified sgRNAs. Mitochondria were isolated and DELE1 levels were analyzed by immunoblotting.

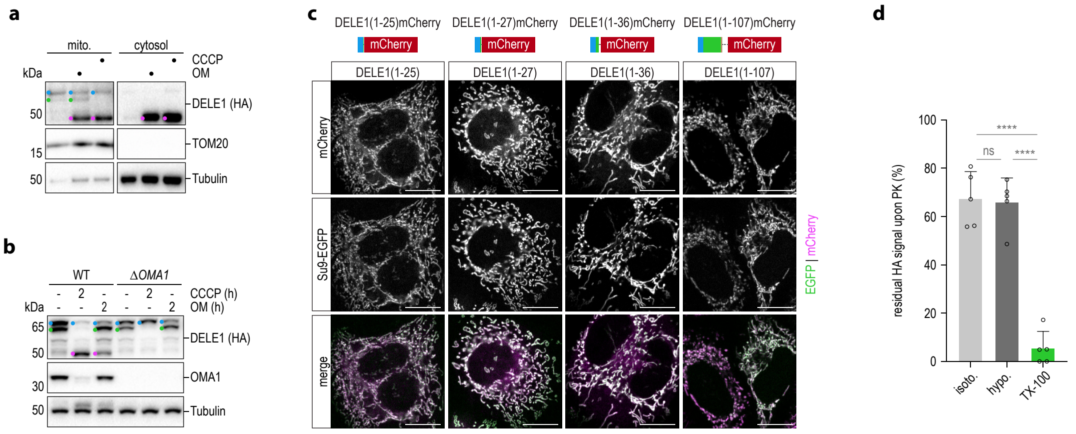

Supplementary Figure 2. DELE1 gives rise to multiple protein species and contains a short presequence that promotes mitochondrial targeting.

a 293T *DELE1*^HA cells were treated with CCCP or OM for 6h followed by isolation of mitochondria (mito.). Localization of DELE1 species to mitochondria or the cytosol was analyzed by immunoblotting.

b 293T wild-type (WT) and OMA1 knockout (Δ*OMA1*) cells stably expressing DELE1-HA were treated with CCCP or OM for 2h and DELE1 processing was analyzed by immunoblotting.

c HeLa cells were transiently transfected with the indicated constructs alongside Su9-EGFP and localization of mCherry and EGFP was analyzed by confocal microscopy in living cells. Scale bars, 10 µm. EGFP (green), mCherry (pink).

d Quantification of the sensitivity of DELE1 to proteinase K under the respective buffer conditions (isotonic buffer (isoto.), osmotic swelling by hypotonic buffer (hypo.), or lysis with TX-100) in mitochondria isolated from HeLa *DELE1*^HA cells. Graph depicts mean residual HA signal after exposure to proteinase K compared to untreated ± s.d. of n=5 independent experiments. Statistical significance was assessed using ordinary one-way ANOVA with Tukey's multiple comparisons correction. ****$P <$ 0.0001, ns = 0.9703.

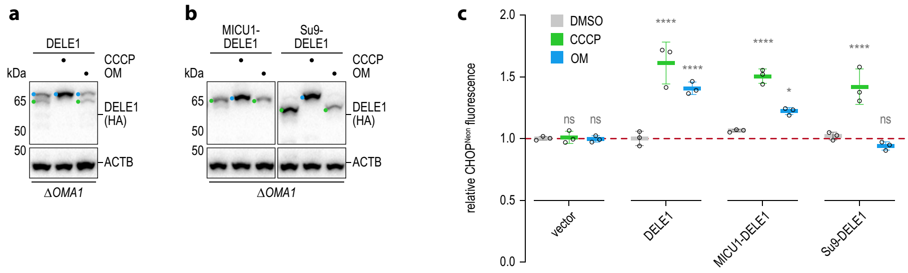

Supplementary Figure 3. Behavior of different DELE1 proteins in the context of mitochondrial perturbations.

a, b 293T OMA1 knockout (Δ*OMA1*) cells were transiently transfected with DELE1 (**a**) or the specified variant (**b**), treated as indicated for 6h and analyzed by immunoblotting.

c $\text{CHOP}^{\text{Neon}}$ fluorescence was analyzed by flow cytometry in HAP1 $\text{CHOP}^{\text{Neon}}$ DELE1 knockout cells upon transient transfection of the indicated constructs or empty vector together with mCherry and subsequent treatments for 8h. $\text{CHOP}^{\text{Neon}}$ fluorescence is shown as fold change vs. the respective untreated control (red line) and statistical significance of CCCP/OM-treated cells compared to DMSO controls was assessed using two-way ANOVA with Dunnett's multiple comparisons correction. Graph depicts mean ± s.d. of n=3 independent experiments. ns, non-significant. ****$P < 0.0001$, *$P = 0.0266$, ns ≥ 0.3361.

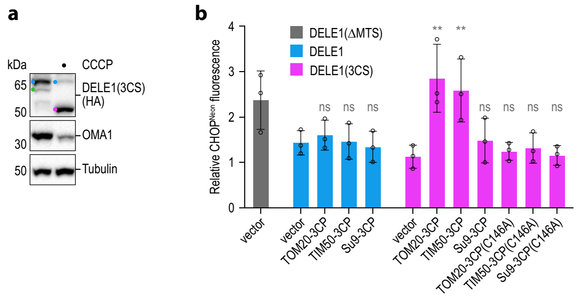

Supplementary Figure 4. Cleavage of DELE1 by OMA1 and 3C protease.

a HeLa wild-type cells were transiently transfected with DELE1(3CS) and empty vector control. 2h before harvest, cells were treated with DMSO or CCCP. Cleavage of the DELE1 protein was analyzed by immunoblotting.

b HAP1 CHOP^{Neon} Δ*OMA1* cells were transiently transfected with the indicated constructs together with mCherry and CHOP^{Neon} fluorescence was analyzed by flow cytometry. Relative CHOP^{Neon} fluorescence to empty vector transfected cells is shown. Graph depicts mean ± s.d. of n=3 independent experiments. DELE1(ΔMTS) serves as control. Statistical significance within DELE1 constructs compared to the respective vector control was assessed by ordinary one-way ANOVA and Dunnett's multiple comparisons correction. DELE1: ns ≥ 0.8711; DELE1(3CS): $^{**}P = 0.0025$ (TOM20-3CP), $^{**}P = 0.0093$ (TIM50-3CP), ns ≥ 0.8567.

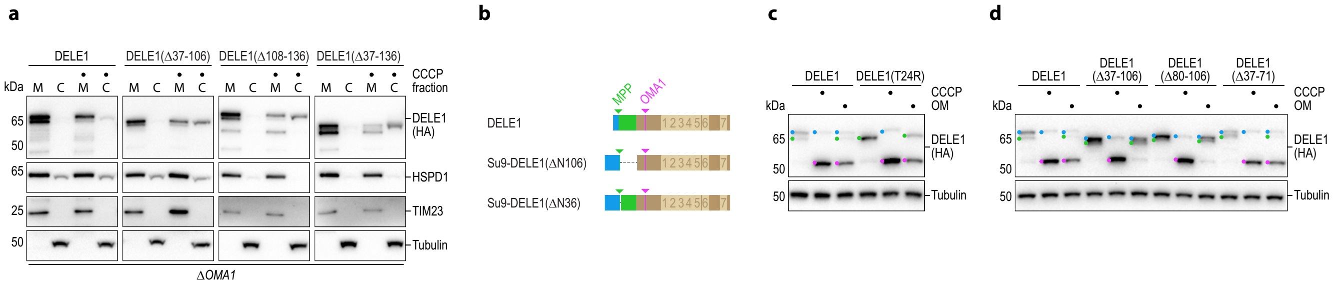

Supplementary Figure 5. Effect of cis elements in the DELE1 MTS in stressed mitochondria.

a HeLa Δ*OMA1* cells stably expressing the indicated MTS variants were treated with CCCP for 2h and localization of DELE1 was analyzed by subcellular fractionation. M, mitochondria. C, cytosol.

b Schematic depicting DELE1 variants used in Fig. 5e with only the DELE1 presequence or the entire MTS replaced by the sorting signals of Su9.

c, d 293T Δ*DELE1* cells were transiently transfected with the indicated constructs, treated with CCCP or OM for 4h and assayed for DELE1 cleavage by immunoblotting. DELE1(Δ37-71) lacks the portion of the MTS immediately adjacent to the presequence but contains the critical stretch between amino acids 80 and 106.

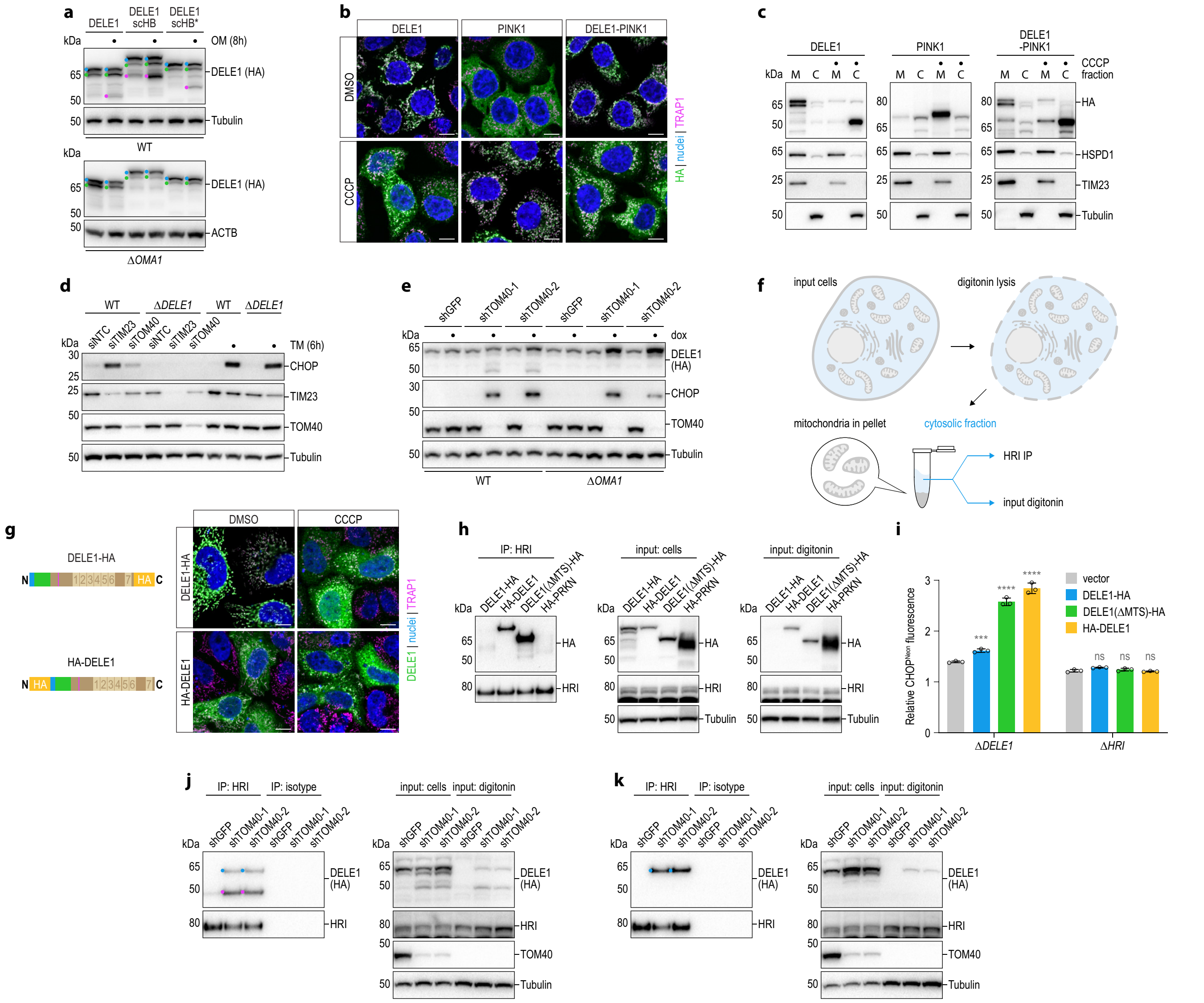

Supplementary Figure 6. Response of DELE1 to different types of mitochondrial protein import stress.
a HeLa wild-type (top) or Δ*OMA1* cells (bottom) were transiently transfected with the specified DELE1 variants and treated as indicated. Processing of the DELE1 protein was analyzed by immunoblotting; scHB, *Saccharomyces cerevisiae* heme-binding domain. scHB*, destabilized HB domain. **b** HeLa cells stably expressing the indicated constructs were treated with CCCP for 4h and localization of HA-tagged proteins was analyzed by confocal microscopy. Scale bars, 10 µm. Nuclei (DAPI, blue), mitochondria (TRAP1, pink), HA (green). **c** 293T cells stably expressing the indicated constructs were treated as in Fig. 6c and analyzed by subcellular fractionation. M, mitochondria. C, cytosol. **d** 293T WT and DELE1 knockout (Δ*DELE1*) cells were transfected with the indicated siRNAs and CHOP levels were analyzed 4 days post transfection or after 6h of tunicamycin (TM) treatment by immunoblotting. **e** TOM40 depletion was induced in HeLa *DELE1*^{HA} WT and OMA1 knockout (Δ*OMA1*) cells with doxycycline (dox) for 6 days and CHOP induction was analyzed by immunoblotting. **f** Schematic depicting lysis procedure for immunoprecipitations using digitonin to specifically analyze protein-protein interactions that take place in the cytosol. **g** HeLa cells were transiently transfected with DELE1 tagged N- or C-terminally with HA and localization was analyzed by confocal microscopy. Scale bars, 10 µm. Nuclei (DAPI, blue), mitochondria (TRAP1, pink), DELE1 (HA, green). **h** Transient expression of the indicated constructs in 293T Δ*OMA1* cells followed by cell lysis using digitonin as detergent. Cytosolic DELE1 species were able to co-precipitate with endogenous HRI as analyzed by immunoblotting. PRKN was used as negative control. **i** Cytosolic DELE1 species can induce CHOP^{Neon} fluorescence in HAP1 CHOP^{Neon} DELE1 knockout (Δ*DELE1*) but not HRI knockout (Δ*HRI*) cells. Cells were transiently transfected with the indicated constructs alongside mCherry and CHOP^{Neon} fluorescence was analyzed 32h after transfection by flow cytometry. Mean ± s.d. of n=3 independent biological samples (two-way ANOVA with Tukey's multiple comparisons correction). ***P = 0.0008, ****P < 0.0001, ns ≥ 0.7528. **j, k** TOM40 knockdown by two different shRNAs was induced in HeLa *DELE1*^{HA} OMA1-proficient (**j**) or OMA1-deficient (**k**) cells for 5 days (shGFP serves as control). Cells were lysed with the digitonin detergent and the co-precipitation of cytosolic DELE1 species with endogenous HRI was analyzed by immunoblotting.

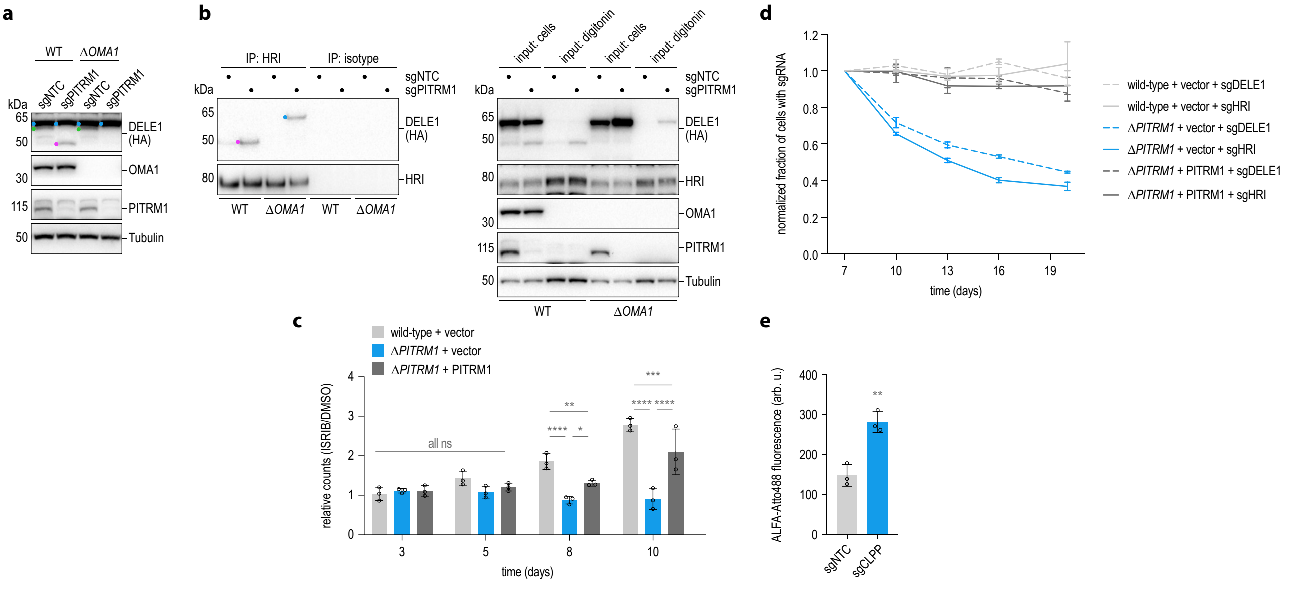

Supplementary Figure 7. ISR signaling in PITRM1 knockout cells promotes cellular fitness.

a HeLa *DELE1*^HA cells were treated as in Fig. 7a and analyzed for DELE1 processing by immunoblotting.

b HeLa *DELE1*^HA cells proficient or deficient for OMA1 were exposed to the indicated sgRNAs and assayed for interaction of endogenous HRI with cytosolic DELE1 species by digitonin lysis as in Supplementary Fig. 6j, k.

c HAP1 *DELE1*^ALFA cells of the indicated genotypes were cultured in medium containing ISRIB or DMSO. Cells were passaged and counted as indicated. Mean ± s.d. of n=3 independent biological replicates (separately seeded and cultured wells for each genotype and treatment) is shown. Statistical significance was assessed using two-way ANOVA with Tukey's multiple comparisons correction. Day 3: ns ≥ 0.8807; Day 5: ns ≥ 0.0983; Day 8: ****P < 0.0001, **P = 0.0057, *P = 0.0331; Day 10: ****P < 0.0001, ***P = 0.0007.

d HAP1 *DELE1*^ALFA cells of the indicated genotypes were infected with lentivirus encoding sgRNAs directed at the specified genes or a non-targeting control together with tRFP. The percentage of tRFP-positive, sgDELE1 or sgHRI-containing cells was normalized to the non-targeting control and to day 7 for each cell line and followed over time. Mean ± s.d. of n=3 independent biological replicates (separately cultured wells for each sgRNA and genotype) is shown.

e DELE1 levels in HAP1 *DELE1*^ALFA cells exposed to the indicated sgRNAs were analyzed by flow cytometry. Graph depicts mean ± s.d. of n=3 independent biological samples. Significance was assessed using a two-tailed Student's *t*-test. **P = 0.0036. Arb. u., arbitrary units.

Supplementary Table 1. Oligonucleotides.

Table of oligonucleotides in 5' to 3' orientation. NTC, non-targeting control; sgRNA, single guide RNA; shRNA short hairpin RNA.

Target	Number	Sequence (5' to 3')
altMIEF	C872.5	AAACAATTGGCCACCATGGCCCCGTGGAGCCGAG
altMIEF	C873.3	TTTGGATCCAATGATCCTCCCCAATTTGCCGTTGAG
CLPP sgRNA	S569.5	CACCGCAGGGCTGTTGATGTACATG
CLPP sgRNA	S570.3	AAACCATGTACATCAACAGCCCTGC
CLUH sgRNA	S346.5	CACCGATCAGTCATGCTCTTAAACG
CLUH sgRNA	S347.3	AAACCGTTTAAGAGCATGACTGATC
DELE1	C372.5	TAGGAATTCGCCACCATGTGGCGCCTCCCGGG
DELE1	C373.3	CTTGGATCCGCCAAAACCTAGTCTTACAACACTC
DELE1 sgRNA	S340.5	CACCGAGCGACATGTGGCGCCTCCC
DELE1 sgRNA	S341.3	AAACGGGAGGCGCCACATGTCGCTC
DELE1 sgRNA	S372.5	CACCGTGCCCGCTCTGGCCCCTAAG
DELE1 sgRNA	S373.3	AAACCTTAGGGGCCAGAGCGGGCAC
DELE1 sgRNA	S416.5	CACCGCTTACAACACTCCTTTCCAG
DELE1 sgRNA	S417.3	AAACCTGGAAAGGAGTGTTGTAAGC
DELE1 sgRNA	S659.5	CACCGCAGTAGTAGCATCGAGTCCG
DELE1 sgRNA	S660.3	AAACCGGACTCGATGCTACTACTGC
EIF2AK1 sgRNA	S342.5	CACCGCCATCGACTTTCCCGCCGA
EIF2AK1 sgRNA	S343.3	AAACTCGGCGGGAAAGTCGATGGC
GFP shRNA	S810.5	CCGGCTATATCATGGCCGACAAGCACTCGAGTGCTTGTCGGCCATGATATAGTTTTTG
GFP shRNA	S811.3	AATTCAAAAACTATATCATGGCCGACAAGCACTCGAGTGCTTGTCGGCCATGATATAG
IMMP1L sgRNA	S477.5	CACCGAGTATAGCCAACAAGTCGAA
IMMP1L sgRNA	S478.3	AAACTTCGACTTGTTGGCTATACTC
IMMP2L sgRNA	S479.5	CACCGCTTGGATCGGGTCGCCTGTG
IMMP2L sgRNA	S480.3	AAACCACAGGCGACCCGATCCAAGC
MICU1	C654.5	AAAGAATTCGCCACCATGTTTCGTCTGAACTCACTTTCTGCTTTGG
MICU1	C655.3	TTTGGATCCAGCGGCTGGAGATTCTGCATGGGCCCTCTTCC
NTC sgRNA	S273.5	CACCGCCAGTACCCAAAAAGCGGG
NTC sgRNA	S274.3	AAACCCCGCTTTTTGGGTACTGGC
NTC sgRNA	S81.5	CACCGGTATGTCGGGAACCTCTCC
NTC sgRNA	S82.3	AAACGGAGAGGTTCCCGACATACC
OMA1 sgRNA	S344.5	CACCGACCGGAGCAGCTTGAAACCG
OMA1 sgRNA	S345.3	AAACCGGTTTCAAGCTGCTCCGGTC
OPA1	C817.5	TTTGAATTCGCCACCATGTGGCGACTACGTCGG
OPA1	C818.3	TTTGGATCCTGACACCTTTCTAAAATGCTTGTCAC
PITRM1	C780.5	TATGCTAGCAATTGGCCACCATGTGGCGCTGCGGCGGGCGG
PITRM1	C781.3	TATGGATCCTTGGATGATCCAGGATGGGTCCTTGGC
PITRM1 sgRNA	S571.5	CACCGAGGAGCCAGGTATTTACACC
PITRM1 sgRNA	S572.3	AAACGGTGTAAATACCTGGCTCCTC
PMPCB sgRNA	S475.5	CACCGAACAGATTAAGAAGTACAC
PMPCB sgRNA	S476.3	AAACGTGTACTTCTTAATCTGTTC
SEC13	C218.5	TTTGAATTCGCCACCATGGTGTCAGTAATTAACACTGTGGATACC
SEC13	C219.3	TTTAAGCTTCTGCTCGTTCTGCTGGCCCTCTGT
TIM50	C652.5	AAAGAATTCGCCACCATGGCGGCCTCGGCAGCGGTGTTC
TIM50	C653.3	AAAGGATCCGCCGCCAGACCCACCTCCGGGCTGTTTGGAGCGAGGCCACAAGC
TOM20	C194.5	TTTGAATTCGCCACCATGGTGGGTCGGAACAGCGCC
TOM20	C195.3	TCAGAAGACTAAGCTTTTCCACATCATCTTCAGCCAAGC

Supplementary Table 2. Antibodies used in this study.
Table of antibodies used in this study with catalogue (cat.) numbers, vendors, applications and dilutions. FACS, flow cytometry; WB, Western blot; IF, immunofluorescence; IP, immunoprecipitation.

Antigen	Cat. number	Vendor	Application	Dilution
FluoTag®-X2 anti-ALFA Atto488	N1502-At488-500µL	NanoTag	FACS	1:500
FluoTag®-X2 anti-ALFA for Western Blotting	N1502-HRP	NanoTag	WB	1:500
Chop	2895T	CST	WB	1:1000
FLAG	F7425	Sigma-Aldrich	WB	1:1000
HA	901514	BioLegend	WB, IF	1:1000, 1:500
PITRM1	10101-2-AP	ProteinTech	WB	1:1000
CLUH / eIF3X	NB100-93306	Novus Biologicals	WB	1:2000
PMPCB	16064-1-A	ProteinTech	WB	1:1000
OMA1	95473S	CST	WB	1:1000
EIF2AK1	20499-1-AP	ProteinTech	WB, IP	1:1000, 1:1000
alpha Tubulin	66031-1-Ig	ProteinTech	WB	1:10000
HSPD1	12165	CST	WB	1:1000
TRAP1	92345	CST	WB, IF	1:1000, 1:200
TIM23	sc-514463	SantaCruz	WB	1:1000
TOMM40	18409-1-AP	ProteinTech	WB	1:2000
Tom20	sc-17764	SantaCruz	WB	1:500
Smac/Diablo	15108	CST	WB	1:1000
ß-actin-HRP (ACTB)	sc-47778	SantaCruz	WB	1:3000
GAPDH	60004-1-Ig	ProteinTech	WB	1:50000
mCherry	26765-1-AP	ProteinTech	IP	1:1150
Goat-anti-Mouse IgG-HRP	170-6516	BioRad	WB	1:3000
Goat-anti-Rabbit IgG-HRP	170-6515	BioRad	WB	1:3000
Alexa Fluor 488-labeled Goat anti-mouse	A11001	Thermo Fisher Scientific	IF	1:500
Alexa Fluor 568-labeled Goat anti-rabbit	A11036	Thermo Fisher Scientific	IF	1:500
