# Supplementary material for: DELE1 tracks perturbed protein import and processing in human mitochondria
Source: Nat Commun. 2022 Apr 6;13:1853. doi: 10.1038/s41467-022-29479-y (PMC8986780; doi:10.1038/s41467-022-29479-y)
Supplement: Supplementary file 3 — Description of Additional Supplementary Files [file 41467_2022_29479_MOESM3_ESM.pdf]

Description of Additional Supplementary Files

File name: Supplementary Data 1

Description: **Results of haploid genetic screen for DELE1 regulators.**

Table listing genes and corresponding mutations identified in mutagenized HAP1 DELE1Alfa cells. A two-sided Fisher's exact test was used to calculate enrichment of mutations in the high or low channel. P-values were FDR-corrected using the Benjamini–Hochberg method.
