# Supplementary material for: DELE1 tracks perturbed protein import and processing in human mitochondria
Source: Nat Commun. 2022 Apr 6;13:1853. doi: 10.1038/s41467-022-29479-y (PMC8986780; doi:10.1038/s41467-022-29479-y)
Supplement: Supplementary file 5 — Reporting Summary [file 41467_2022_29479_MOESM5_ESM.pdf]

nature portfolio

Corresponding author(s): Evelyn Fessler
Lucas T. Jae

Last updated by author(s): 27.02.2022

Reporting Summary

Nature Portfolio wishes to improve the reproducibility of the work that we publish. This form provides structure for consistency and transparency in reporting. For further information on Nature Portfolio policies, see our Editorial Policies and the Editorial Policy Checklist.

Statistics

For all statistical analyses, confirm that the following items are present in the figure legend, table legend, main text, or Methods section.

n/a	Confirmed	
☐	☒	The exact sample size (n) for each experimental group/condition, given as a discrete number and unit of measurement
☐	☒	A statement on whether measurements were taken from distinct samples or whether the same sample was measured repeatedly
☐	☒	The statistical test(s) used AND whether they are one- or two-sided<br>*Only common tests should be described solely by name; describe more complex techniques in the Methods section.*
☒	☐	A description of all covariates tested
☐	☒	A description of any assumptions or corrections, such as tests of normality and adjustment for multiple comparisons
☐	☒	A full description of the statistical parameters including central tendency (e.g. means) or other basic estimates (e.g. regression coefficient) AND variation (e.g. standard deviation) or associated estimates of uncertainty (e.g. confidence intervals)
☐	☒	For null hypothesis testing, the test statistic (e.g. F, t, r) with confidence intervals, effect sizes, degrees of freedom and P value noted<br>*Give P values as exact values whenever suitable.*
☒	☐	For Bayesian analysis, information on the choice of priors and Markov chain Monte Carlo settings
☒	☐	For hierarchical and complex designs, identification of the appropriate level for tests and full reporting of outcomes
☒	☐	Estimates of effect sizes (e.g. Cohen's d, Pearson's r), indicating how they were calculated

Our web collection on statistics for biologists contains articles on many of the points above.

Software and code

Policy information about availability of computer code

Data collection	For data collection the following software was used: BD FACSDiva 8.0.1 and 8.0.2, LasX 3.7.0.20979, ZEN 2009 5,5,0,443, Image Lab 5.2, SPARKCONTROL v3.1
Data analysis	Calculations of statistical significance were performed using GraphPad Prism version 9.3.0. For data analysis the following software was used: FlowJo 10.8, BD FACSDiva 8.0.1 and 8.0.2, GraphPad Prism 9.3.0, Image Lab version 6.1.0, Fiji 2.0.0-rc-69/1.52p, Bowtie 1.0.1, intersectBED v2.26.0, UniProt/clustalo 1.2.4

For manuscripts utilizing custom algorithms or software that are central to the research but not yet described in published literature, software must be made available to editors and reviewers. We strongly encourage code deposition in a community repository (e.g. GitHub). See the Nature Portfolio guidelines for submitting code & software for further information.

Data

Policy information about availability of data

All manuscripts must include a data availability statement. This statement should provide the following information, where applicable:

- Accession codes, unique identifiers, or web links for publicly available datasets
- A description of any restrictions on data availability
- For clinical datasets or third party data, please ensure that the statement adheres to our policy

The data supporting the findings of this study are available in the article and its supplementary information. Source data are provided with this paper. Original immunoblot images and plot data generated in this study are provided in the Source Data file. Sequencing datasets have been deposited at the NCBI Sequence Read Archive under the accession code PRJNA750901 [https://www.ncbi.nlm.nih.gov/sra/?term=PRJNA750901] and are publicly available. The corresponding processed

data are provided in Supplementary Data 1. Human genome 19 (hg19) is publicly available through University of California Santa Cruz [https://genome.ucsc.edu/index.html]. Data are also available from the corresponding authors upon reasonable request.

Field-specific reporting

Please select the one below that is the best fit for your research. If you are not sure, read the appropriate sections before making your selection.

☒ Life sciences ☐ Behavioural & social sciences ☐ Ecological, evolutionary & environmental sciences

For a reference copy of the document with all sections, see nature.com/documents/nr-reporting-summary-flat.pdf

Life sciences study design

All studies must disclose on these points even when the disclosure is negative.

Sample size	No sample size calculation was performed, as the study does not involve animals or human subjects. Sample sizes were chosen based on previous experience and common practice in the field. Individual specimen correspond to culture wells that contain thousands to millions of single cells. For the haploid genetic screen a total of n=2.4x10^7 single cells were analyzed. Flow cytometry experiments included n=3 biological replicates. Experiments were independently reproduced as detailed in the 'Methods' section and below.
Data exclusions	No data were excluded.
Replication	All experiments were independently repeated and were reliably reproduced. Additionally, multiple cell lines were used to confirm reproducibility of the findings. For the haploid genetic screen (Fig. 1f) a total of n=2.4x10^7 single cells were analyzed. Data are representative of the following numbers of independent experiments: Fig. 1a, n=2; Fig. 1c, n=3; Fig. 1d, n=3; Fig. 1e, n=3; Fig. 1g, n=3; Fig. 1h, n=4; Fig. 1i, n=3; Fig. 1j, n=2; Fig. 1k, n=2; Fig. 2a, n=2; Fig. 2b, n=3; Fig. 2c, n=3; Fig. 2e, n=3; Fig. 2f, n=2; Fig. 2i, n=2; Fig. 2j, n=3; Fig. 2k, n=3; Fig. 2l, n=5; Fig. 3a, n=3; Fig. 3b, n=3; Fig. 3c, n=3; Fig. 3e, n=3; Fig. 3f, n=2; Fig. 3g, n=3; Fig. 4b, n=3; Fig. 4c-d, n=3; Fig. 4e, n=5; Fig. 4f, n=4; Fig. 5b, n=2; Fig. 5c, n=2; Fig. 5d, n=3; Fig. 5e, n=3; Fig. 5f, n=3; Fig. 5g, n=3; Fig. 6a, n=3; Fig. 6b, n=3; Fig. 6c, n=2; Fig. 6d, n=4 (d3), n=2 (d5); Fig. 6e, n=3 (d3), n=4 (d4); Fig. 7a, n=3; Fig. 7b, n=2; Fig. 7c, n=4; Fig. 7d, n=4; Fig. 7e, n=3; Fig. 7f, n=2; Fig. 7g, n=4; Supplementary Fig. 1b, n=3; Supplementary Fig. 1c, n=2; Supplementary Fig. 2a, n=3; Supplementary Fig. 2b, n=4; Supplementary Fig. 2c, n=2; Supplementary Fig. 2d, n=5; Supplementary Fig. 3a, n=2; Supplementary Fig. 3b, n=2; Supplementary Fig. 3c, n=3; Supplementary Fig. 4a, n=3; Supplementary Fig. 4b, n=3; Supplementary Fig. 5a, n=2; Supplementary Fig. 5c, n=3; Supplementary Fig. 5d, n=3; Supplementary Fig. 6a, n=2; Supplementary Fig. 6b, n=3; Supplementary Fig. 6c, n=3; Supplementary Fig. 6d, n=2; Supplementary Fig. 6e, n=2; Supplementary Fig. 6g, n=2; Supplementary Fig. 6h, n=2; Supplementary Fig. 6i, n=3; Supplementary Fig. 6j, n=2; Supplementary Fig. 6k, n=2; Supplementary Fig. 7a, n=4; Supplementary Fig. 7b, n=4; Supplementary Fig. 7c, n=2; Supplementary Fig. 7d, n=4; Supplementary Fig. 7e, n=2.
Randomization	Randomization was not relevant to this study, all tests were in-vitro. Cells were clearly identified based on genotype and/or treatment. Cells were cultured under identical conditions and unbiasedly allocated to well positions and treatments. Samples were harvested, processed and analyzed in random order when possible.
Blinding	Blinding was not relevant to this study, as all tests were in-vitro and no subjective rating of data was performed. Experimental conditions were typically evident from the data and parameters such as location of well positions is fixed. Key observations of microscopy-based assays were orthogonally analyzed by quantitative methods that report population averages (e.g. fractionation, flow cytometry).

Reporting for specific materials, systems and methods

We require information from authors about some types of materials, experimental systems and methods used in many studies. Here, indicate whether each material, system or method listed is relevant to your study. If you are not sure if a list item applies to your research, read the appropriate section before selecting a response.

Materials & experimental systems

n/a	Involved in the study	
☐	☒	Antibodies
☐	☒	Eukaryotic cell lines
☒	☐	Palaeontology and archaeology
☒	☐	Animals and other organisms
☒	☐	Human research participants
☒	☐	Clinical data
☒	☐	Dual use research of concern

Methods

n/a	Involved in the study	
☒	☐	ChIP-seq
☐	☒	Flow cytometry
☒	☐	MRI-based neuroimaging

Antibodies

Antibodies used	Antibodies used in this study are listed in Supplementary Table S2.<br>antigen | clone number | cat. number | lot number | vendor | application | dilution<br>FluoTag®-X2 anti-ALFA Atto488 | 1G5 | N1502-At488-500µL | 15190101 | NanoTag | FACS | 1:500<br>FluoTag®-X2 anti-ALFA for Western Blotting | 1G5 | N1502-HRP | 15200103 | NanoTag | WB | 1:500

Chop | L63F7 | 2895T | 12 | CST | WB | 1:1000
FLAG | - | F7425 | 086M4803V | Sigma-Aldrich | WB | 1:1000
HA | 16B12 | 901514 | n/a | BioLegend | WB, IF | 1:1000, 1:500
PITRM1 | - | 10101-2-AP | ProteinTech | WB | 1:1000
CLUH / eIF3X | NB100-93306 | - | Novus Biologicals | WB | 1:2000
PMPCB | - | 16064-1-A | - | ProteinTech | WB | 1:1000
OMA1 | D4J7K | 95473S | 1 | CST | WB| 1:1000
EIF2AK1 | - | 20499-1-AP | 00013826 | ProteinTech | WB, IP | 1:1000, 1:1000
alpha Tubulin | 1E4C11 | 66031-1-Ig | - | ProteinTech | WB | 1:10000
Smac/Diablo | D5S3R | 15108 | 1 | CST | WB | 1:1000
HSPD1 | D6F1 | 12165 | 3 | CST | WB | 1:1000
mNEON | 32F6 | 32f6-10 | 70117021AB | Chromotek | FACS | 1:2500
TRAP1 | D3D7N | 92345 | 1 | CST | WB, IF | 1:1000, 1:200
TIM23 | H-8 | sc-514463 | B0717 | SantaCruz | WB | 1:1000
TOMM40 | - | 18409-1-AP | - | ProteinTech | WB | 1:2000
TOM20 | F-10| sc-17764 | J1218 | SantaCruz | WB | 1:500
ß-actin-HRP (ACTB) | C4 | sc-47778 | K1418 | SantaCruz | WB | 1:3000
GAPDH | 1E6D9 | 60004-1-Ig | - | ProteinTech | WB | 1:50000
mCherry | - | 26765-1-AP | - | ProteinTech | IP | 1:1150
Goat-anti-Mouse IgG-HRP | - | 170-6516 | n/a | BioRad | WB | 1:3000
Goat-anti-Rabbit IgG-HRP | - | 170-6515 | n/a| BioRad | WB | 1:3000
Alexa Fluor 488-labeled Goat anti-mouse | - | A11001 | 1834337 | Thermo Fisher Scientific | FACS, IF | 1:500
Alexa Fluor 568-labeled Goat anti-rabbit | - | A11036 | 1832035 | Thermo Fisher Scientific | IF | 1:500

Validation

For immunoblot analysis, the correct size of the protein of interest was assessed by protein marker and for microscopy, the localization of the stained protein was assessed.
The specificity of the following antibodies was tested using knockout/knockdown cell lines:
OMA1 (CST 95473S)
TRAP1 (CST 92345)
PITRM1 (ProteinTech 10101-2-AP)
CLUH / eIF3X (Novus Biologicals NB100-93306)
PMPCB (ProteinTech 16064-1-A)
TIM23 (SantaCruz sc-514463)
TOM40 (ProteinTech 18409-1-AP)
The anti EIF2AK1 antibody (ProteinTech 20499-1-AP) was used for immunoprecipitation of endogenous HRI protein; the identity of the precipitated proteins was assessed by mass-spectrometry, identifying EIF2AK1 among the most abundant proteins.
For validation by the manufacturer please see the following websites:
ALFA https://nano-tag.com/products/fluotag-x2-anti-alfa
Chop https://en.cellsignal.de/products/primary-antibodies/chop-l63f7-mouse-mab/2895?site-searchtype=Products&N=4294956287&Ntt=2895t&fromPage=plp&_requestid=2307582
Flag https://www.sigmaaldrich.com/DE/de/product/sigma/f7425
HA https://www.biolegend.com/en-us/products/purified-anti-ha-11-epitope-tag-antibody-11374
PITRM1 https://www.ptglab.com/products/PITRM1-Antibody-10101-2-AP.htm
CLUH / eIF3X https://www.novusbio.com/products/eif3x-antibody_nb100-93306
PMPCB https://www.ptglab.com/products/PMPCB-Antibody-16064-1-AP.htm
OMA1 https://en.cellsignal.de/products/primary-antibodies/oma1-d4j7k-rabbit-mab/95473?site-searchtype=Products&N=4294956287&Ntt=95473s&fromPage=plp&_requestid=2310100
EIF2AK1 https://www.ptglab.com/products/EIF2AK1-Antibody-20499-1-AP.htm
alpha Tubulin https://www.ptglab.com/products/tubulin-Alpha-Antibody-66031-1-Ig.htm
Smac/Diablo https://en.cellsignal.de/products/primary-antibodies/smac-diablo-d5s3r-rabbit-mab/15108
HSPD1 https://en.cellsignal.de/products/primary-antibodies/hsp60-d6f1-xp-rabbit-mab/12165
mNEON https://www.chromotek.com/products/detail/product-detail/mneongreen-antibody-32f6/
TRAP1 https://en.cellsignal.de/products/primary-antibodies/trap1-hsp75-d3d7n-rabbit-mab/92345
TIM23 https://www.scbt.com/p/tim23-antibody-h-8
TOM40 https://www.ptglab.com/products/TOMM40-Antibody-18409-1-AP.htm
TOM20 https://www.scbt.com/de/p/tom20-antibody-f-10?requestFrom=search
ß-actin-HRP (ACTB) https://www.scbt.com/p/beta-actin-antibody-c4?requestFrom=search
GAPDH https://www.ptglab.com/products/GAPDH-Antibody-60004-1-Ig.htm
mCherry https://www.ptglab.com/products/mCherry-Antibody-26765-1-AP.htm

Eukaryotic cell lines

Policy information about cell lines

Cell line source(s)

Cells lines obtained from authenticated stocks were a kind gift from the Brummelkamp laboratory (HEK293T, HeLa; obtained from ATCC). HAP1 cell line was generated in the Burmmelkamp laboratory (Carette et al., Nature 2011).

Authentication

Purchased cell lines were authenticated by source. During cultivation, cell lines were authenticated by monitoring phenotypic features, such as morphology and growth characteristics.

Mycoplasma contamination

All cell lines tested negative for mycoplasma.

Commonly misidentified lines
(See ICLAC register)

No commonly misidentified cell lines were used in this study.

Flow Cytometry

Plots

Confirm that:

☒ The axis labels state the marker and fluorochrome used (e.g. CD4-FITC).

☒ The axis scales are clearly visible. Include numbers along axes only for bottom left plot of group (a 'group' is an analysis of identical markers).

☒ All plots are contour plots with outliers or pseudocolor plots.

☒ A numerical value for number of cells or percentage (with statistics) is provided.

Methodology

Sample preparation	Cells were acquired and cultured according to Methods section. Before analysis, cells were trypsinized, homogenized and vortexed.
Instrument	BD LSRFortessa (BD, Franklin Lakes, New Jersey, USA), BD Fusion
Software	Data were analyzed using BD FACSDiva (BD, Franklin Lakes, New Jersey, USA) or FlowJo software (TreeStar Inc, Ashland, Oregon, USA).
Cell population abundance	The gates used for sorting were set to top 4% DELE1-ALFA high and bottom 4% DELE1-ALFA low. Supplementary Figure 1a details the gating strategy and cell population abundance.
Gating strategy	For analytical flow cytometry or sorting of living cells, cells were first gated on FSC-A vs SSC-A to exclude debris. Subsequently, single cells were gated using the FSC-H vs FSC-W blot. Single cells were analyzed for fluorescence.<br>Cell sorting of fixed cells for mutation mapping: cells were identified using the FCS-A vs SSC-A blot to exclude debris. Subsequently, single cells were identified using DAPI-A vs DAPI-W blot. Single cells were displayed in a histogram, on which gating on 1n DNA content was performed to exclude diploid cells. Sorting was performed on the haploid cell population, the gates used for sorting were set to top 4% DELE1-ALFA high and bottom 4% DELE1-ALFA low.

☒ Tick this box to confirm that a figure exemplifying the gating strategy is provided in the Supplementary Information.
