# Supplementary material for: DELE1 tracks perturbed protein import and processing in human mitochondria
Source: Nat Commun. 2022 Apr 6;13:1853. doi: 10.1038/s41467-022-29479-y (PMC8986780; doi:10.1038/s41467-022-29479-y)
Supplement: Supplementary file 6 — Source Data [file 41467_2022_29479_MOESM6_ESM.zip › Source Data/Uncropped immunoblot source data.pdf]

Uncropped immunoblot source data.

Figure 1a

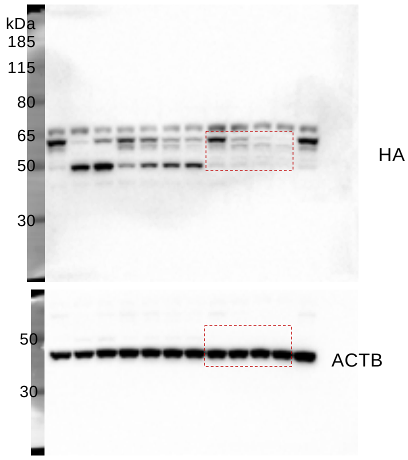

Figure 1d

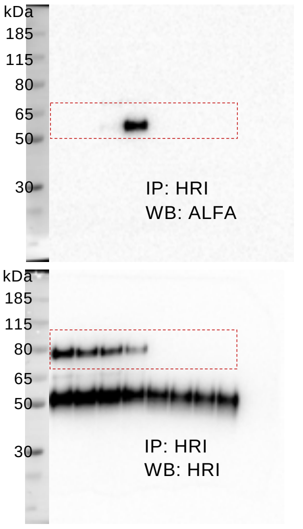

Figure 1e

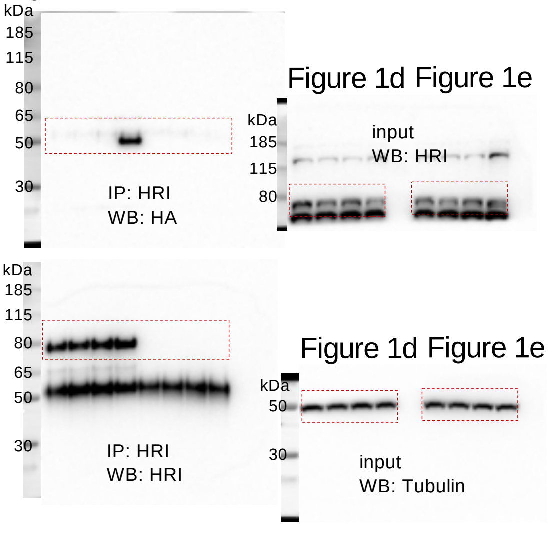

Figure 1g

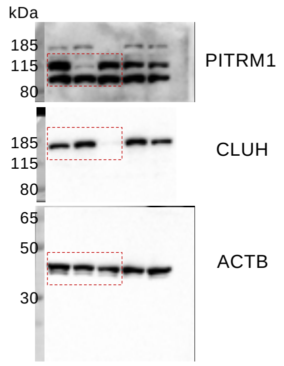

Figure 1i

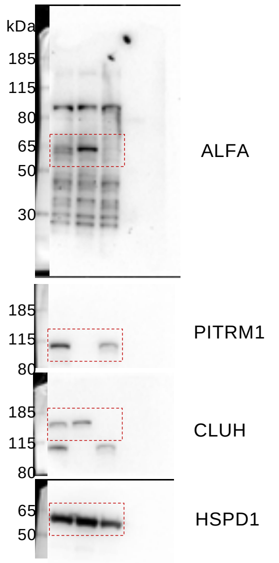

Figure 1j

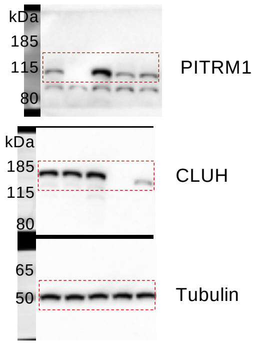

Figure S1b

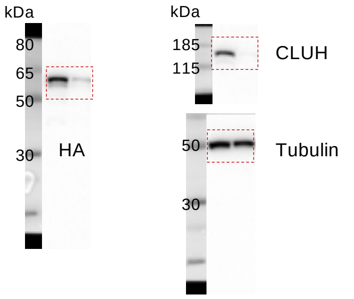

Figure S1c

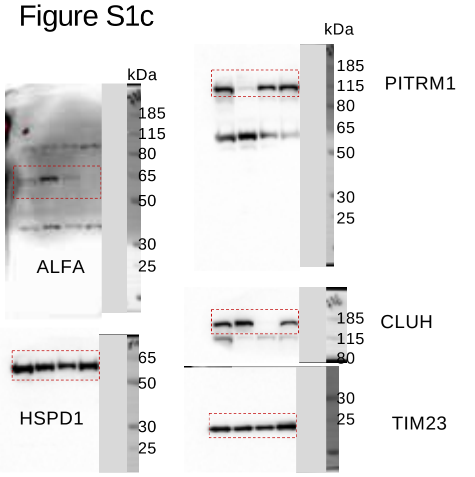

Figure 2a

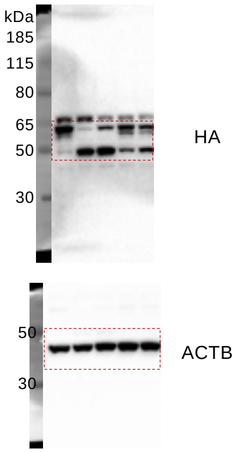

Figure 2b

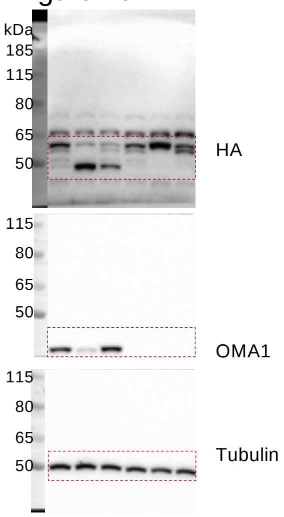

Figure 2c

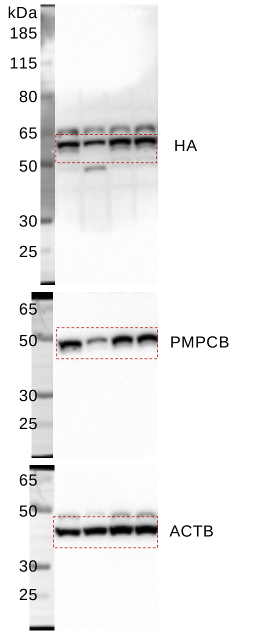

Figure 2e

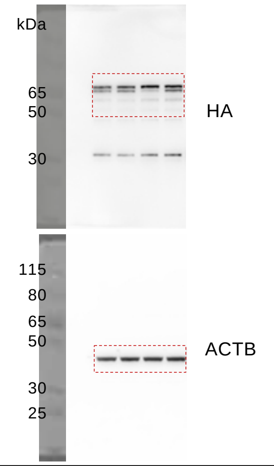

Figure 2l

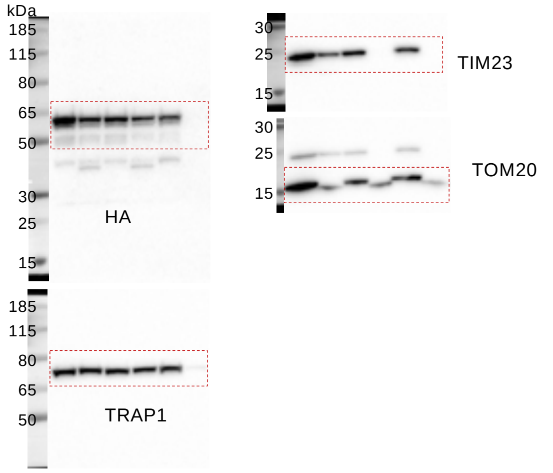

Figure S2a

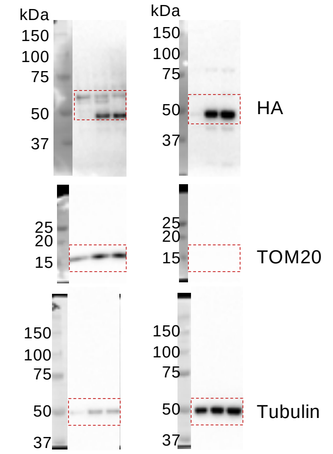

Figure S2b

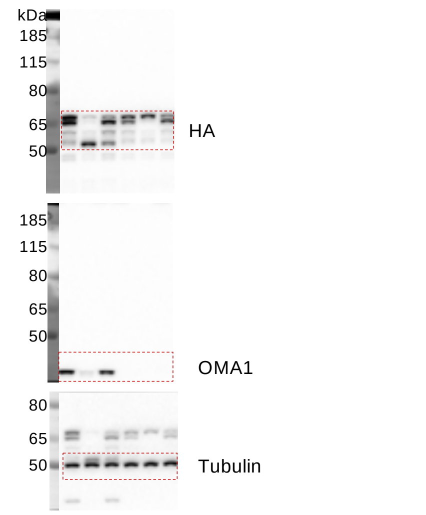

Figure 3a

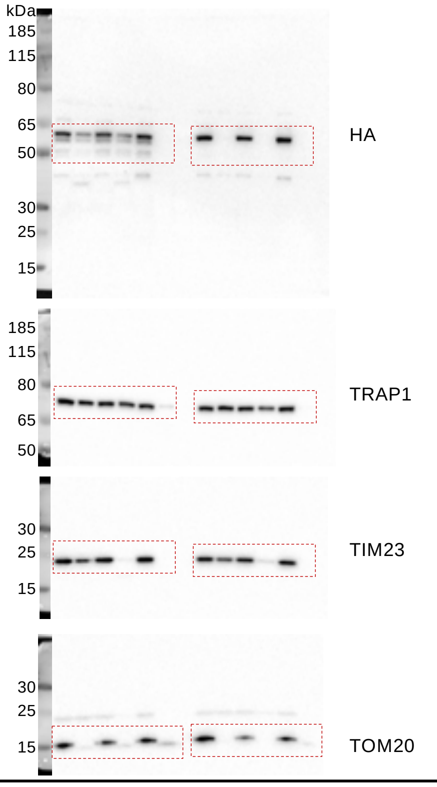

Figure 3b

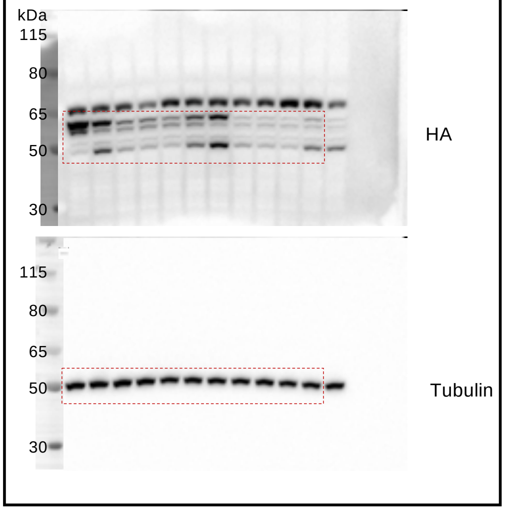

Figure 3c

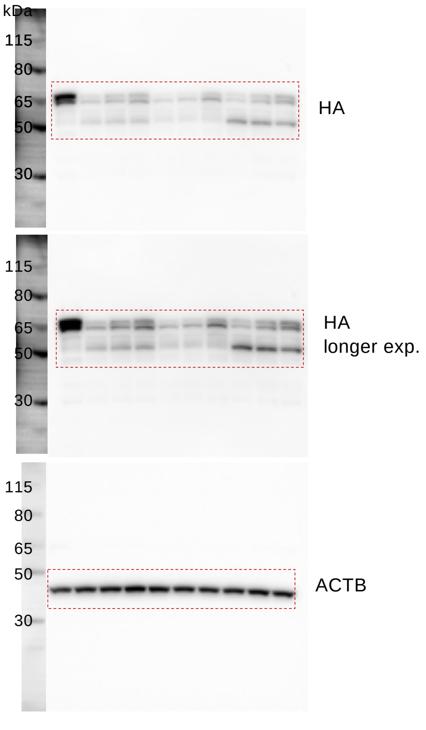

Figure 3g

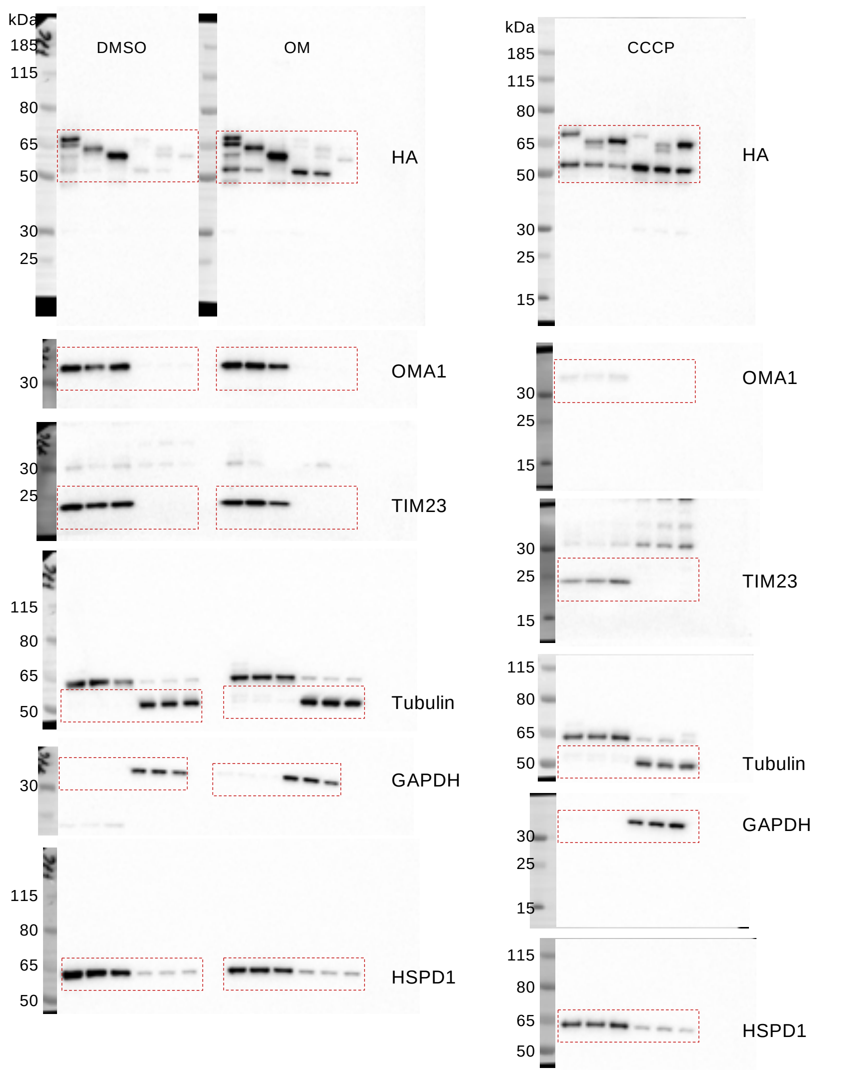

Figure 3e

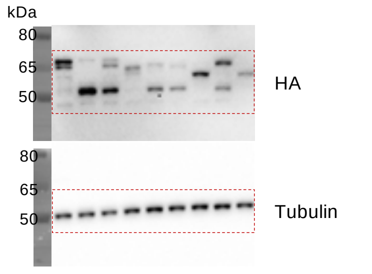

Figure S3a and b

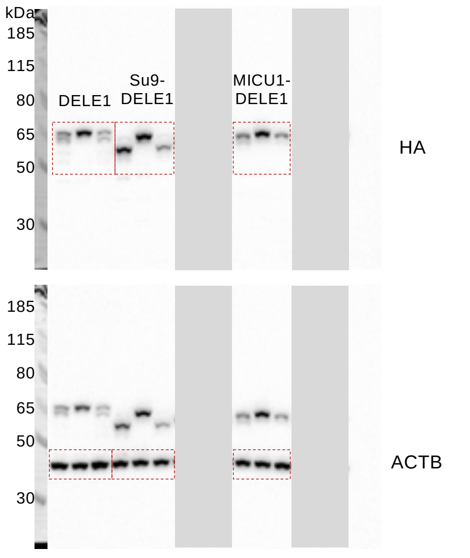

Figure 4b

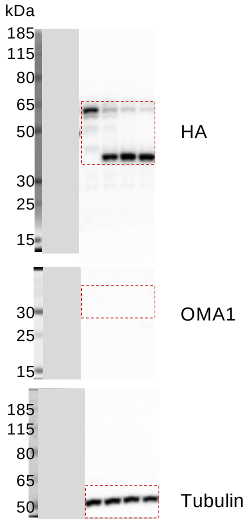

Figure 4f

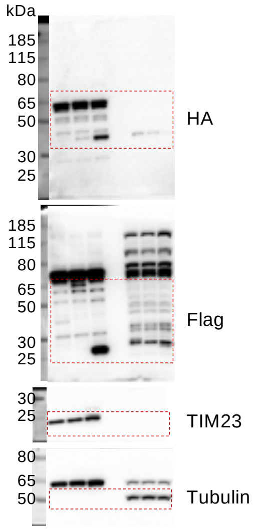

Figure S4a

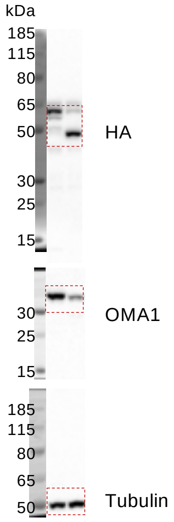

Figure 5b

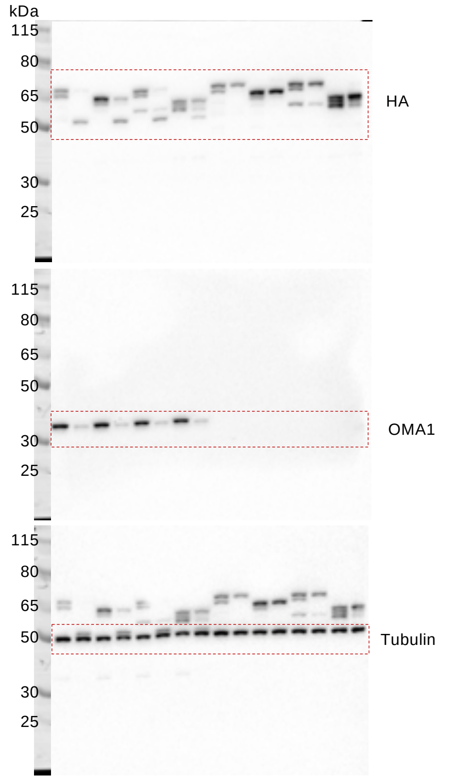

Figure 5d

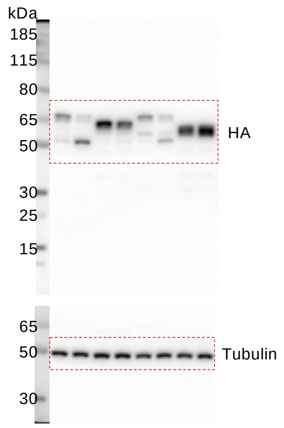

Figure 5e

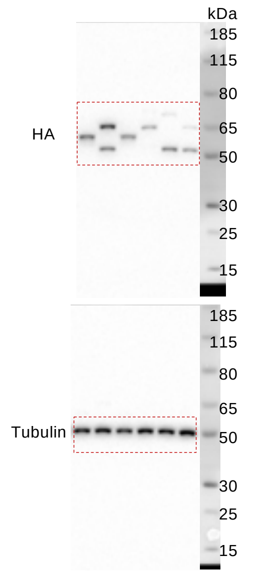

Figure 5f

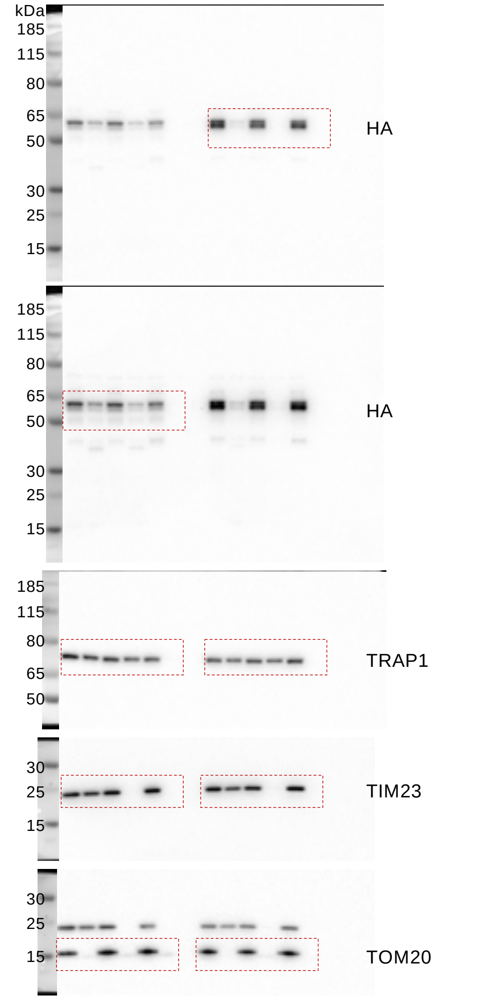

Figure 5g

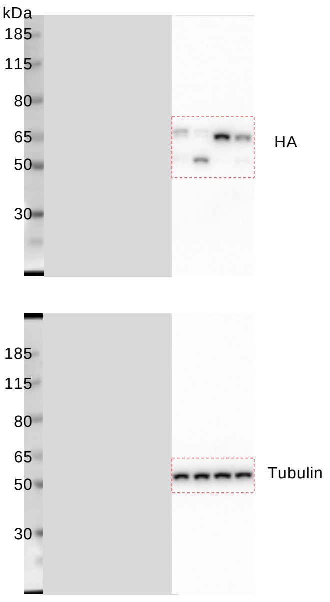

Figure S5a

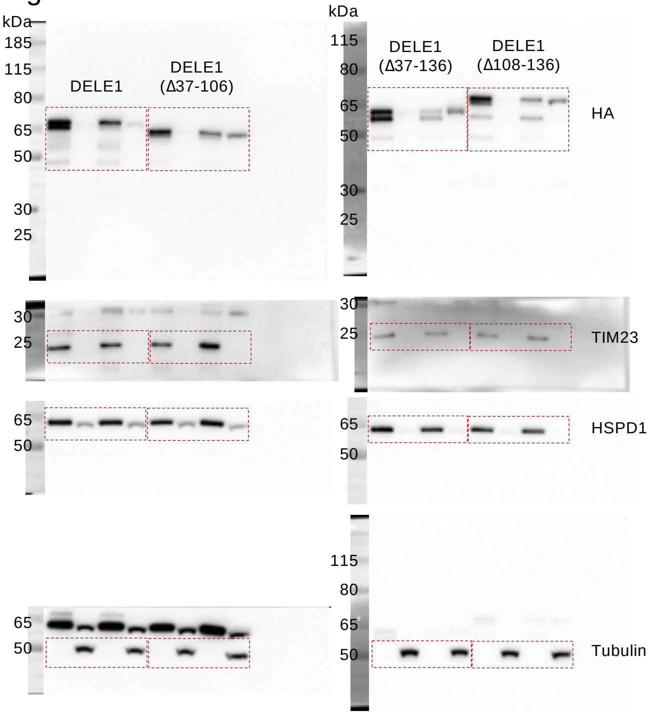

Figure S5c

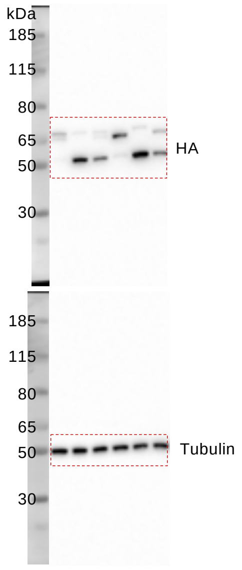

Figure S5d

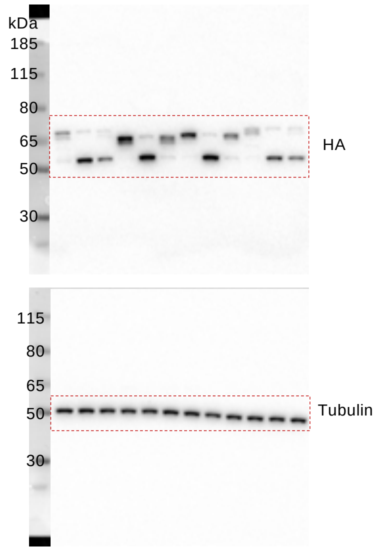

Figure 6a

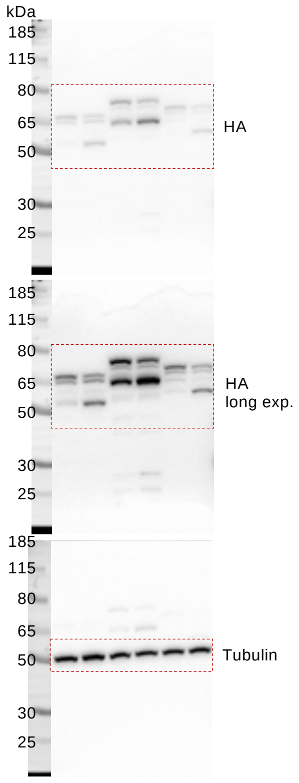

Figure 6d

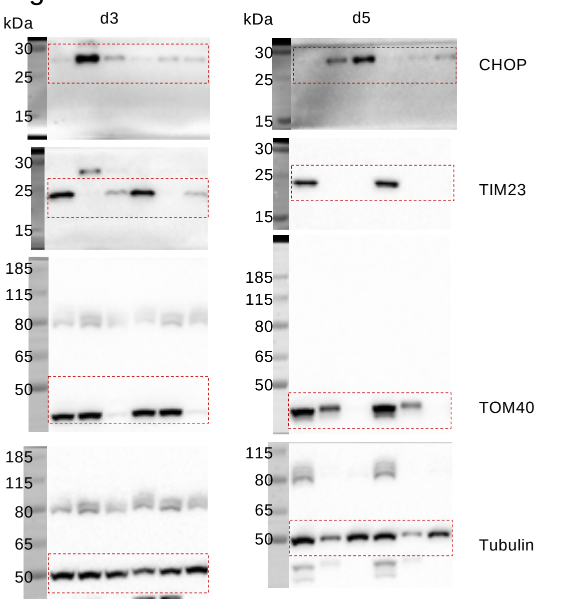

Figure 6e

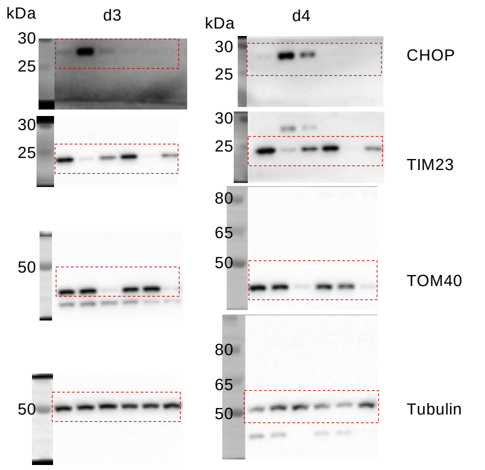

Figure S6c

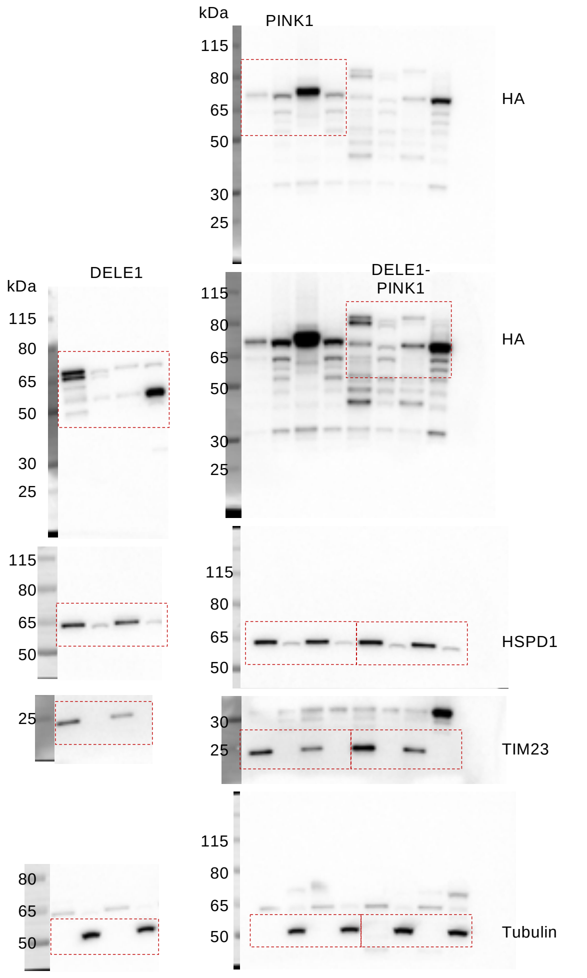

Figure S6a

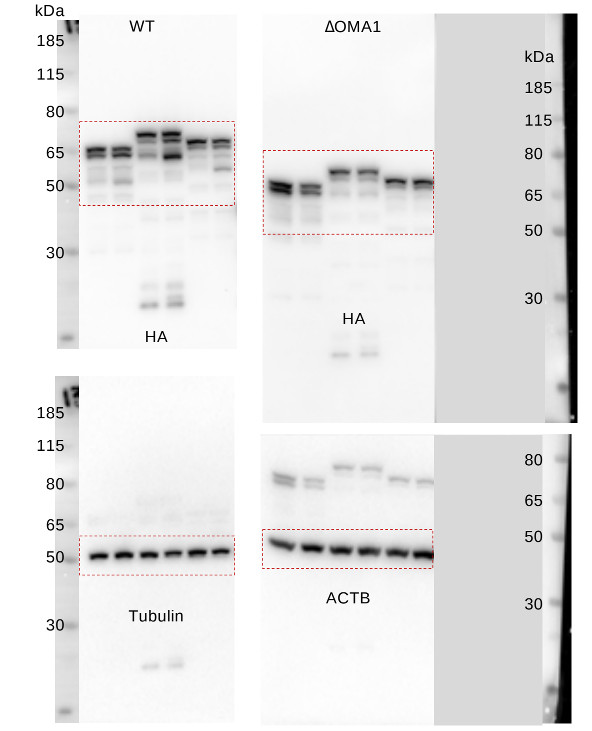

Figure S6d

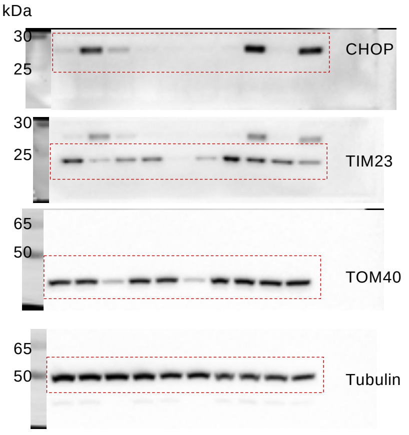

Figure S6e

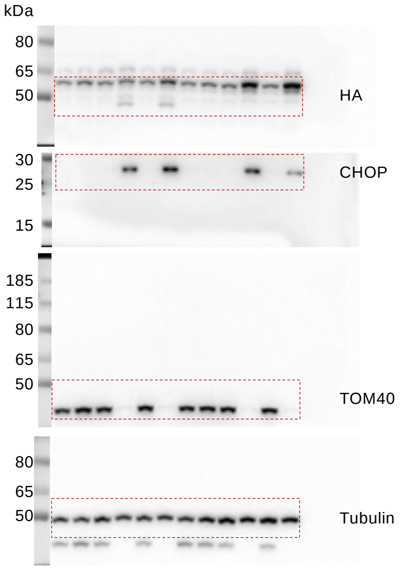

Figure S6h

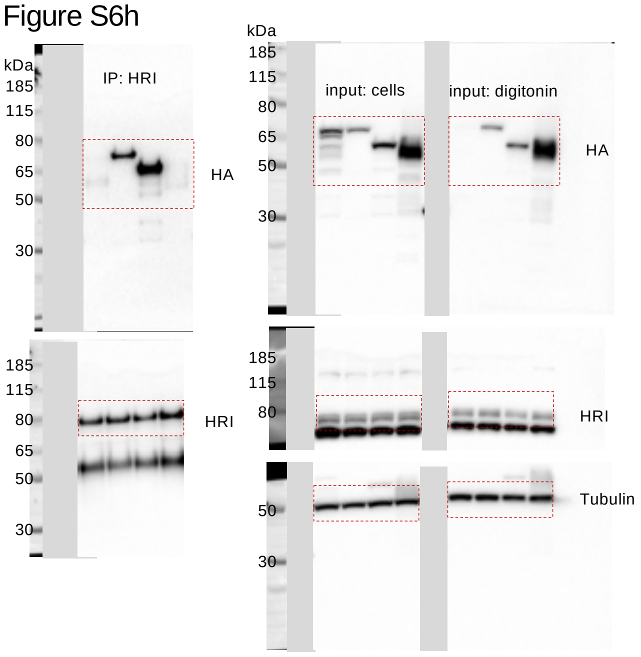

Figure S6j

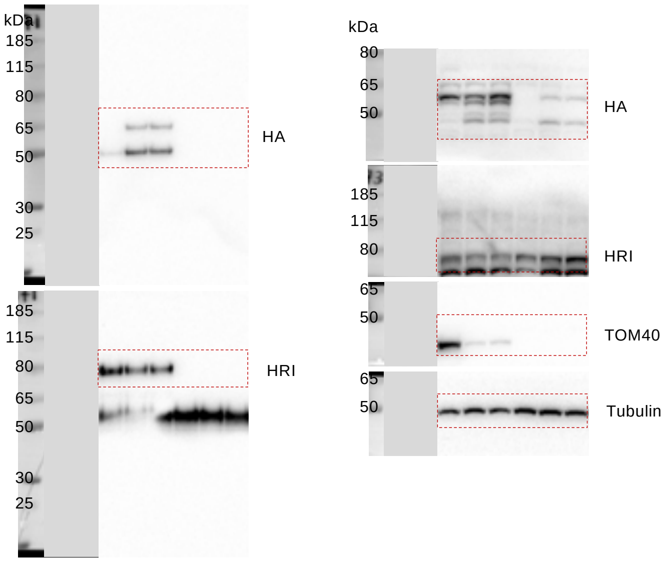

Figure S6k

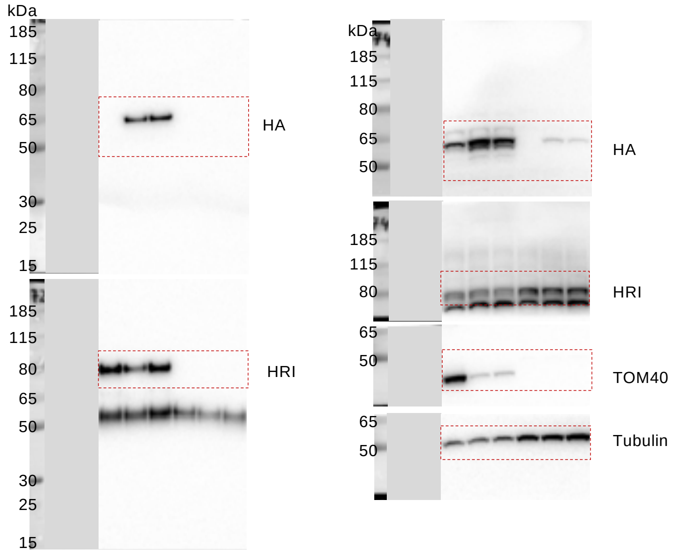

Figure 7a

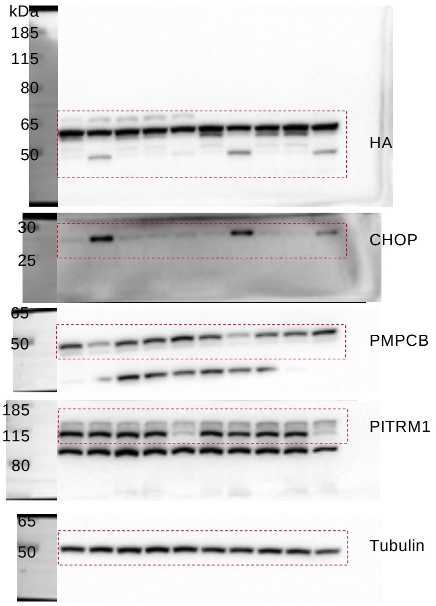

Figure 7b

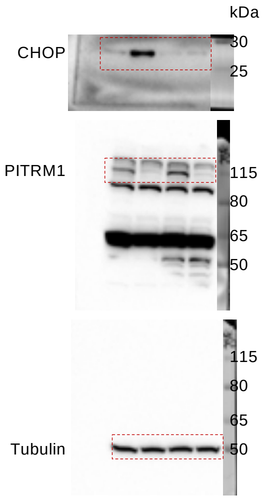

Figure 7c

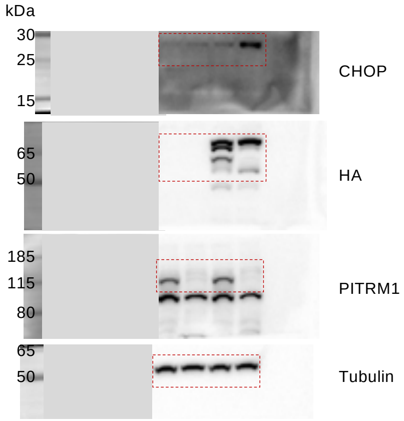

Figure 7d

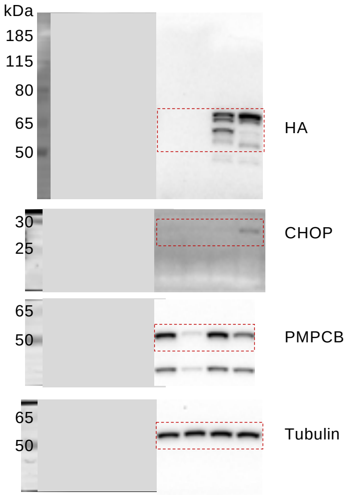

Figure 7e

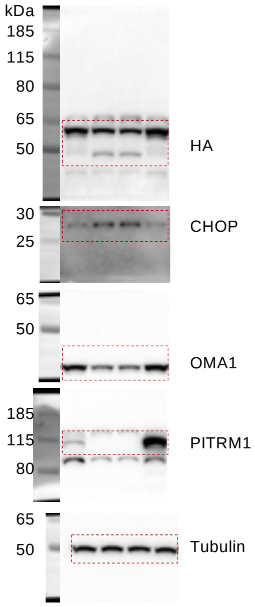

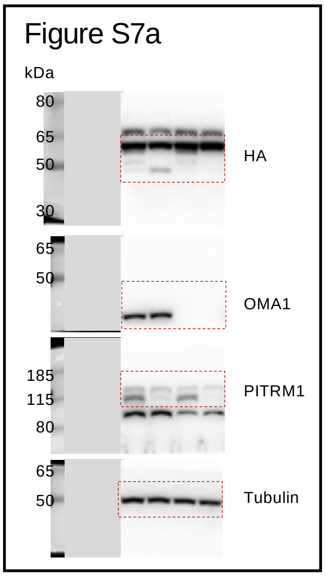
Figure S7a
kDa
80
65
50
30
HA
65
50
OMA1
185
115
80
PITRM1
65
50
Tubulin

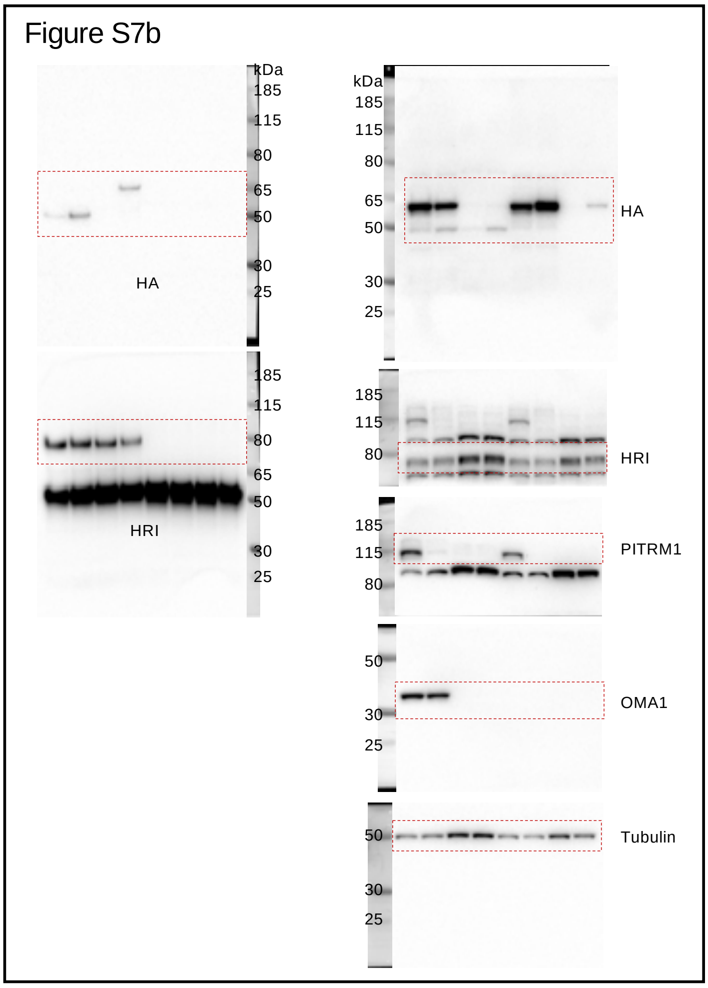
Figure S7b
kDa
185
115
80
65
50
30
25
HA
HRI
HA
HRI
PITRM1
OMA1
Tubulin
